# Influence of artificial aging: mechanical and physicochemical properties of dental composites under static and dynamic compression

**DOI:** 10.1007/s00784-021-04122-0

**Published:** 2021-08-28

**Authors:** D. C. Gornig, R. Maletz, P. Ottl, M. Warkentin

**Affiliations:** 1 Dres. Irina & Thorsten Brandt, Orthodontic Practice, Wiesbaden, Germany; 2 Department of Material Science and Medical Engineering, Faculty of Mechanical Engineering and Marine Technology, University of Rostock, Rostock, Germany; 3 Department of Prosthodontics and Material Science, Faculty of Medicine, University of Rostock, Rostock, Germany

**Keywords:** Artificial aging, Dental composites, Static and dynamic compression tests, Mechanical and physicochemical properties

## Abstract

**Objective:**

The aim of the study was to evaluate the influence of filler content, degradation media and time on the mechanical properties of different dental composites after in vitro aging.

**Materials and Methods:**

Specimens (1 mm^3^) of three commercially available composites (GrandioSO®, Arabesk Top®, Arabesk Flow®) with respect to their filler content were stored in artificial aging media: artificial saliva, ethanol (60%), lactic acid (pH 5) and citric acid (pH 5). Parameters (Vickers microhardness, compressive strength, elastic modulus, water sorption and solubility) were determined in their initial state (control group, *n* = 3 for microhardness, *n* = 5 for the other parameters) and after 14, 30, 90 and 180 days (*n* = 3 for microhardness, *n* = 5 for the other parameters for each composite group, time point and media). Specimens were also characterized with dynamic-mechanical-thermal analysis (compression tests, *F* =  ± 7 N; *f* = 0.5 Hz, 1 Hz and 3.3 Hz; *t* = 0–170 °C).

**Results:**

Incorporation of fillers with more than 80 w% leads to significantly better mechanical properties under static and dynamic compression tests and a better water sorption behavior, even after chemical degradation. The influence of degradation media and time is of subordinate importance for chemical degradation.

**Conclusion:**

Although the investigated composites have a similar matrix, they showed different degradation behavior. Since dentine and enamel occur only in small layer thickness, a test specimen geometry with very small dimensions is recommended for direct comparison. Moreover, the use of compression tests to determine the mechanical parameters for the development of structure-compatible and functionally adapted composites makes sense as an additional standard.

Clinical relevance

Preferential use of highly filled composites for occlusal fillings is recommended.

## Introduction

Dental composites (*DC*) are used as a standard filling material, mostly due to their advantageous functional and esthetic characteristics. In accordance, the adhesive bonding of *DC* allows a cavity design without additional expansion of preparations, which would be essential for filling therapies using amalgam. In light of minimal-invasive dentistry, the absence of macro-retentions leads to purely defect oriented removal of caries and the greatest possible preservation of natural teeth [1]. In a study with 22,391 restorations, 9805 fillings of different materials had to be removed. Reasons for replacing composite fillings were first at all secondary caries (47%), followed by fractures of the restoration (24%). In comparison, 57% of amalgam restorations were replaced due to secondary caries and 25% due to restoration fractures [2]. By virtue of their location in the oral cavity, *DC* are subjected to high requirements. Adequate clinical, mechanical, chemical and physical material properties, biocompatibility and a simple application are essential. *DC* have to resist different mechanical forces and they are exposed to moisture, various temperatures and fluctuating pH values. This leads to aging, which changes the mechanical properties over time. Temperature influences aging processes via increasing reaction rate, which accelerates chemical degradation. Thereby, *DC*-polymers are chemically divided. At the same time, diffusion of oxygen and water (vapor) increases, which additionally enables degradation reactions. In addition, chemical degradation (oxidation or solvolysis) of polymers occurs. Covalent ester bonds of the *DC*-polymer matrix are broken by absorbed water molecules [3]. These can diffuse into *DC*-polymers and accumulate in less densely packed areas due to hydrogen bonds [4]. If *DC* contain hydrophilic components and emulsifiers, they have a higher water sorption than without [5]. Water molecules which are already contained in *DC* also influence further penetration of water. In turn, absorbed water can cause reactions with pigments, fillers and plasticizers due to the soaking process. Therefore, frequent drying and soaking influence the aging process of the specific *DC*-material. Also, physiologically occurring reagents, e.g. acids, bases or enzymes can catalyze hydrolysis [6, 7] and lead to aging. Göpferich postulated that changes in pH by degradation products lead to a reinforcing feedback effect [8]. Accordingly, degradation process of *DC* can be considered to be multifactorial and complex, though the clinical relevance is high as it was shown that the second most common factor for replacement for dental restorations with *DC* was a respective fracture of the filling material [2]. In order to avoid degradation damage and to develop new, degradation-resistant materials, knowledge of factors influencing degradation is necessary. Mechanical failure of *DC* has already been examined in many different ways. Only a few studies considered chemical degradation [9, 10]. Subsequent, dynamic-mechanical tests are missing in these studies. *DC* are heterogeneous polymers which show viscoelastic behavior. Dynamic-mechanical-thermal analysis (*DMTA*) shows both properties of *DC* and aging of polymer matrix very well. The combination of static and dynamic analyses makes studies like this work essential and differentiates it from the existing literature. In comparison to other degradation studies, the selected study design was an in vitro long-term investigation, which combined and compared important influencing factors and allows a comparison to human tooth. Moreover, water sorption and solubility have been investigated in numerous studies [11] without a comparison with mechanical properties. Numerous authors tried to convert the complex process into in vitro models, but there is currently no published literature for composites under compression after artificial aging. A standardized test procedure is absent. However, special techniques have been developed over the past 20 years to mimic the in vivo situation. A large number of test equipment were developed. This includes methods based on articulators and freely rotatable friction wheels as well as devices for reproducing the load patterns of a chewing cycle. In contrast to this work, various authors used chewing simulators to imitate clinically relevant conditions [15, 16]. Nevertheless, these simulators cannot completely imitate the intraoral situation and up to date they are mostly used without different degradation media. Further studies in this area would be desirable. As the prevalence of lesions due to dietary habits, such as extensive consumption of acidic fruit juices and sports drinks, is increasing [17], it is useful to investigate different media. Similar attempts can be found in the literature. Simplified models of the matrix-filler complex [9], the composite-tooth connection [18] or pure composites [19] were examined. Distilled water, saline, ethanol, heptanes, artificial saliva, acetic acid, propane, lactic acid and citric acid were used as degradation media [20–22]. In accordance, the aim of this study was to evaluate the influence of filler content, different storage media and incubation time on mechanical properties and physicochemical characteristics of *DC* under static and dynamic compression tests after in vitro aging.

## Materials and methods

### Composites

**Fig. 1:**
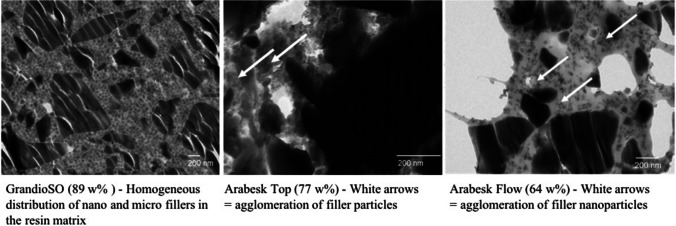
Ultrathin Sect. (90 nm); transmission electron microscopy (TEM) of tested composites

Three commercially available composites were selected with respect to their filler content. One low-filled (< 70 w%; composite C, Arabesk Flow: batch code 1,131,254; VOCO GmbH, Cuxhaven, Germany, shade A3), one medium-filled (71–79 w%; composite B, ArabeskTop: batch code 1,218,277; VOCO GmbH, shade A3) and one highly filled (> 80 w%; composite A, GrandioSO: batch code 1,212,204; VOCO GmbH, shade A3) composite were chosen (Table 1 and Fig. 1).

**Table 1:** Composites chosen by filler content

Composite product name	Batch code	Resin matrix	Filler particles	Filler size	Filler content (w%)
Composite A (GrandioSO)	1,212,204	BisGMA, TEGDMA, BisEMA	Silanized silicon dioxide nanofillers	20–40 nm	89
			Glass ceramic fillers	Ø 1 µm	
Composite B (Arabesk Top)	1,218,277	BisGMA, TEGDMA, UDMA	Highly dispersed silicon dioxide	Ø 0.05 µm	**77**
			Barium-/strontium borosilicate	Ø 0.7 µm	
Composite C (Arabesk Flow)	1,131,254	BisGMA, TEGDMA, UDMA	Highly dispersed silicon dioxide	No manufacturer information	64
			Barium-/strontium borosilicate	Ø 0.7 µm

### Specimen preparation

**Fig. 2:**
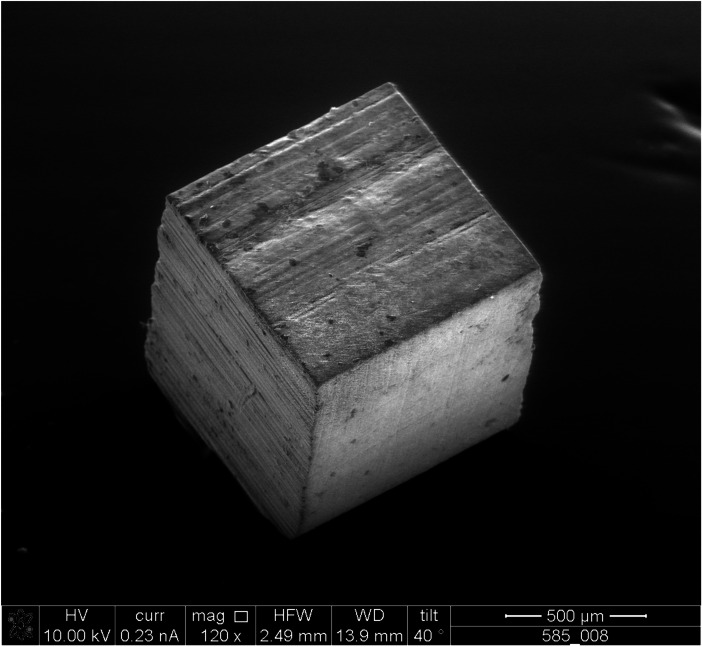
Exemplary SEM-image of specimen (composite A/specimen number 585)

A small specimen geometry of 1 mm^3^ was manufactured for obtaining a comparison between the in vitro results and human teeth. With these samples (Fig. 2), compression tests instead of 3-point-bending were used which is usually recommended by the DIN EN ISO standard [23].

**Fig. 3:**
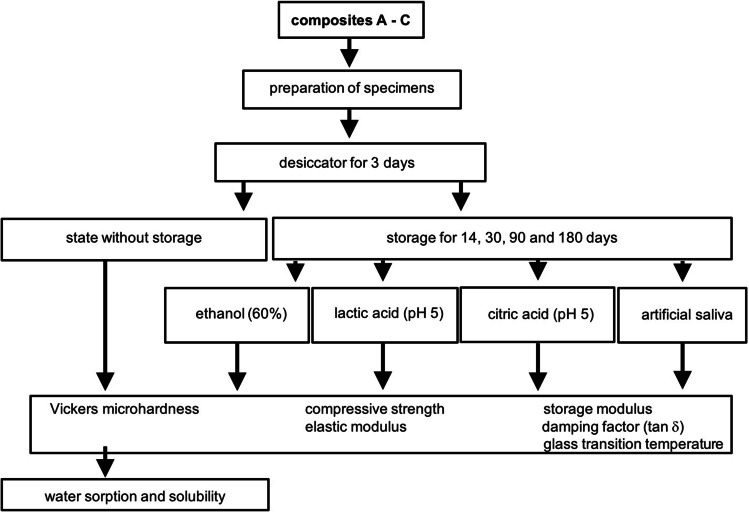
Test procedure

First of all, samples were prepared according to the manufacturer’s guidelines (length: 25 mm, width: 1 mm, height: 1 mm). The composites were placed in a custom-made PTFE mold (polytetrafluoroethylene) and light-cured according to the manufacturer’s recommendation. After curing, the specimens were removed from the mold and supernatants were gradually removed using SiC grinding paper (Microcut SILICON CARBIDE GRINDING PAPER WET OR DRY; Buehler, IL, USA). The surface that was affected by the curing light first was marked as top side. After that procedure, the specimens were stored under exclusion of light for 1 week to ensure post-polymerization. In the next step, specimens were packed airtight in Parafilm (Parafilm M; Karl Hecht GmbH & Co KG, Sondheim, Deutschland) and embedded in epoxy resin (EpoThin 2, Epoxy System; Buehler). The Parafilm avoids a bonding between composite and epoxy resin as well as a resin infiltration into the sample. After the samples had hardened (24 h at room temperature), they were sawn into cubes (1 mm^3^) using a diamond wire saw (Histo-Saw DDM-P216; Medim, Wilmington, USA) with a diamond wire thickness of 200 μm and a diamond size of 40 μm. In the following step, all specimen cubes were randomized. For this purpose, all cubes of each composite were mixed, placed in an Eppendorf reaction vessel (0.5 ml) and numbered. In order to avoid measurement errors due to water sorption, e.g. by air or water cooling of the diamond wire saw, specimens were dried in a desiccator for 3 days before storage in the various media (Fig. 3).

### Artificial aging

Finally, all samples were incubated in the appropriate storage media at 37 °C (Incubator 1000; Heidolph Instruments GmbH & Co. KG, Schwabach, Germany) with continuous agitation (130 rpm) (Unimax 1010; Heidolph Instruments GmbH & Co. KG, Schwabach, Germany). In order to rule out potential bacterial growth during degradation, the storage media of each sample was refreshed completely every 4 weeks.

Artificial saliva represents the simplest physiological influence on composites in form of hydrolysis. The used chemicals are based on the New Recipe Formula Germany (NRF 7.5). Lactic acid is an example of metabolic products of plaque bacteria such as *Lactobacilli *spp. and *Streptococcus* *mutans*, which break down glucose to lactate by fermentation for their own energy generation. The 80% lactic acid (CAS 79–33-4; Carl Roth, Karlsruhe, Deutschland) was pipetted into 100 ml of artificial saliva and set a pH of 5 using a portable pH meter (FE20—FiveEasy pH; Mettler Toledo, Giessen, Germany). Citric acid had been chosen as an example of many acids ingested through food. 19.213 g citric acid (CAS 77–92-9; Carl Roth, Karlsruhe, Deutschland) was added to 100 ml distilled water for preparation of 1 M citric acid. With help of a pH meter (FE20—FiveEasy pH; Mettler Toledo) pH of 5 was adjusted by dilution with artificial saliva. Ethanol (CAS 64–17-5; VWR International GmbH, Darmstadt, Deutschland) was also used as storage medium. The US Food and Drug Administration (*FDA*) defined ethanol as a liquid that simulates fatty foods and alcoholic beverages. Moreover, it has led to degradation of composites in several studies [24, 25]. The “softening” by ethanol is described in the literature [26, 27]. Sixty milliliters of ethanol (100%) and 40 ml of distilled water were mixed up for 100 ml of solution.

### Test methods

**Fig. 4:**
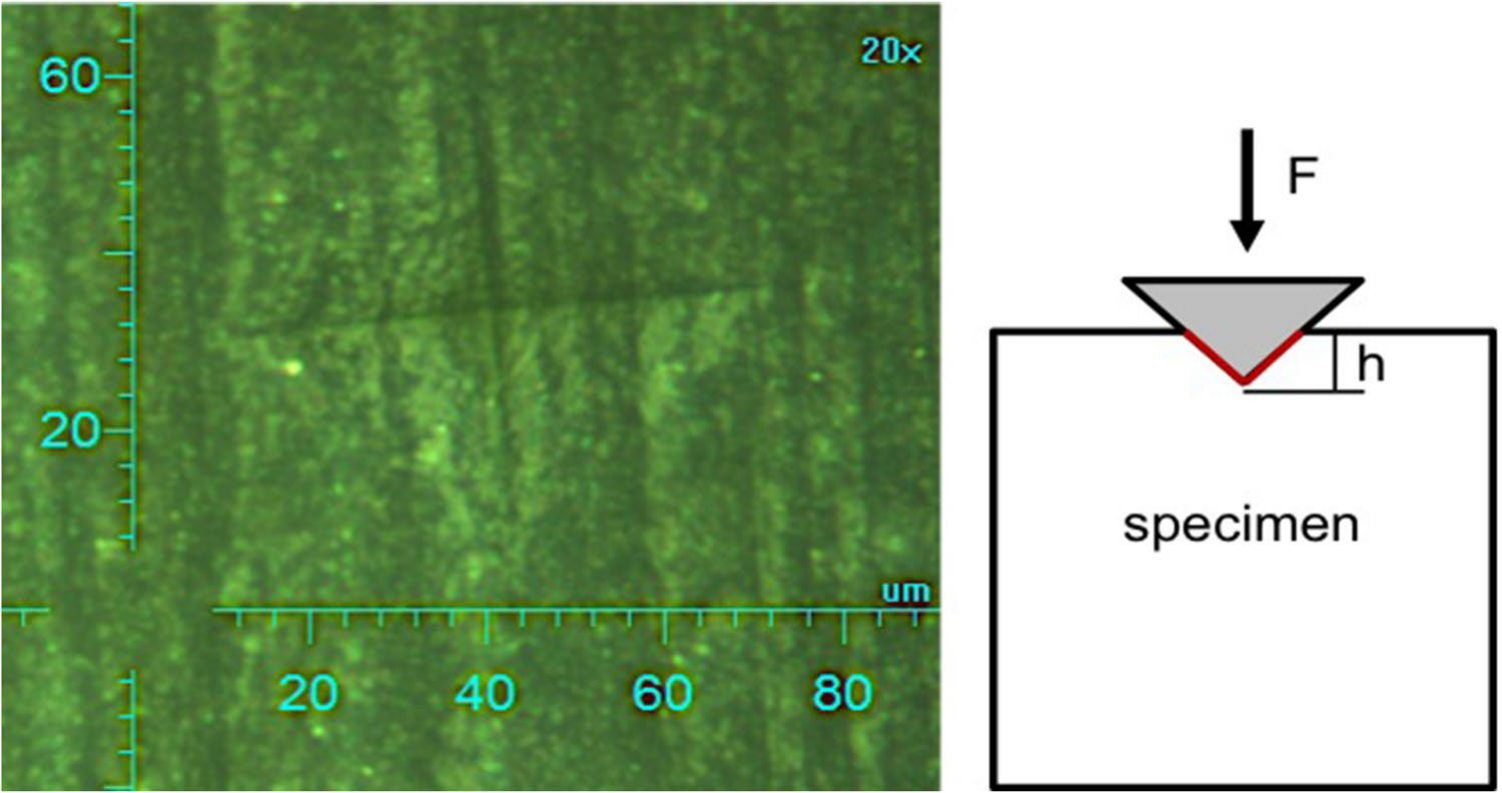
Penetration impression after measurement

Vickers microhardness (*MHV*) was determined in the initial state (control group, *n* = 3 for each composite group) and after 14, 30, 90 and 180 days (*n* = 3 for each composite group, time point and media). It was measured computer controlled with a Fischerscope HM 2000 (HELMUT FISCHER GMBH, Sindelfingen-Maichingen, Germany). A series of indentations (four measuring points on each specimen) were set by using a distance of approximately 300 µm between the measuring points and the border of the specimen. Martens hardness (*HM*) was determined electronically using an equilateral Vickers diamond pyramid by continuously measuring and recording force and depth of the penetration (Fig. 4). A maximum load of 1 N was applied with an indentation rate of 0.2 N s^−1^ and a holding time of 5 s at maximum load. Afterwards, *MHV* was directly calculated by the computer software.

Compressive strength is defined as *σ*_max_ where samples fractured. According to the DIN standard [28], compressive strength and elastic modulus were measured for the initial state (*n* = 5 for each composite group) and after degradation (*n* = 5 for each composite group, time point and media). Specimens were examined with a universal testing machine (BZ2.5/TN1S; Zwick GmbH & Co. KG, Ulm, Germany) under free uniaxial compression load (load cell 2500 N, crosshead speed 1 mm min^−1^) and a preload of 10 N. Force and deformation were recorded electronically using the software TestXpert II (Zwick GmbH & Co. KG, Ulm, Germany). Data were converted in the mechanical parameters Young’s modulus *E*_*C*_ (GPa) and compressive strength *σ*_max_ (MPa).

*DMTA* was used to record the viscoelastic properties of the sample [29]. Therefore, a sinusoidal load was applied to the specimen and this periodic mechanical stress results in a corresponding signal in form of tension or deformation [30]. Specimens were characterized for the initial state (*n* = 5 for each composite group) and after degradation (*n* = 5 for each composite group, time point and media). With *DMTA* storage modulus *E´*, damping factor *tan* *δ* and glass transition temperature *Tg* were determined at three frequencies (0.5 Hz, 1 Hz and 3.3 Hz) in a temperature range from 0 to 170 °C (heating rate 1 K min^−1^). The data of the compression tests were evaluated using software Proteus Analysis (Netzsch Gerätebau GmbH, Selb, Germany).

The mass of the specimens was determined in the initial state (m1; *n* = 3 for each composite), after storage in storage media (m2; *n* = 3 for each composite group, time point and media) and after subsequent drying in the desiccator (m3; *n* = 3 for each composite group, time point and media). With respect to specimen geometry and the resulting low weight, a high-resolution analytical balance with a readability of 0.01 mg and a linearity of <  ± 0.1 mg (Sartorius ME 235S-OCE; Sartorius Weighing Technology GmbH, Göttingen, Germany) was used for precise mass determination. Calculation for water sorption (*Wsp*, formula 1) and solubility (*Wsl*, formula 2) was carried out according to DIN EN ISO 4049 [23] using the following formulas:

1 $$Wsp=\frac{m2-m3}{V}$$

2 $$Wsl=\frac{m1-m3}{V}$$

### Statistics

For statistical data analysis, the experimentally determined data were evaluated using the software program IBM SPSS Statistics 21 (IBM Software Group, Chicago, USA). Kruskal–Wallis test was used as a non-parametric test for independent samples. The median and interquartile range (*IQR*) of the experimental results were calculated and compared. The influence of filler content, degradation media and time on the various parameters was examined. The significance level was rated with *p* < 0.05 as significant. Graphics with box-and-whisker plots with 25/75% percentile were created. In addition, a bivariate Spearman rank correlation analysis (*p* < 0.05) was used to investigate a linear relationship between the characteristic value and the filler content of the composite, with the Spearman-Rho correlation coefficient (*r*_*s*_) describing the strength of the linear relationship.

## Results

### Composite characteristics in initial state—control group

In order to be able to identify and analyze degradation processes, the initial state of the composites before storage in various media was characterized at first hand.

**Fig.5:**
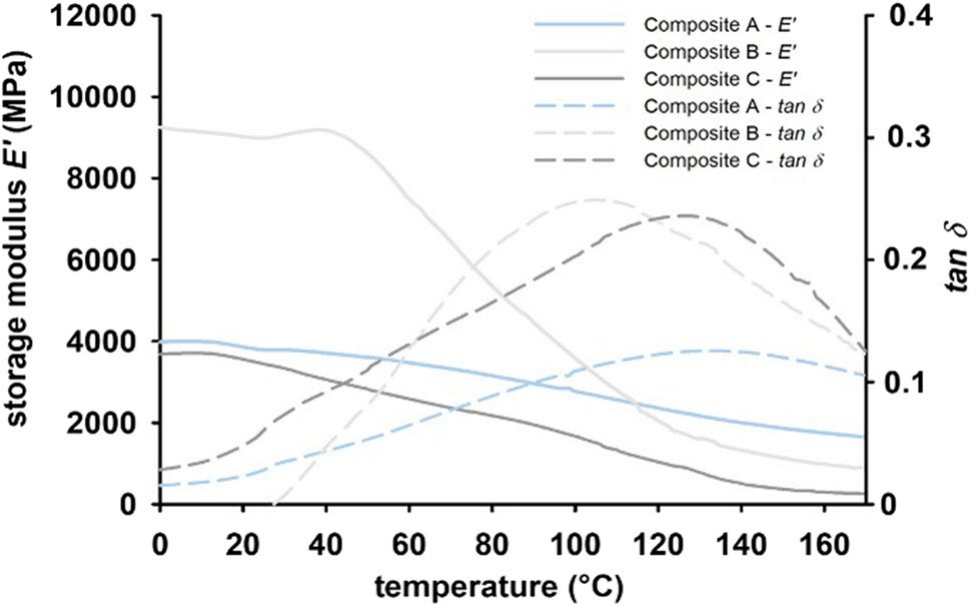
Exemplary representation of frequency-dependent temperature profile of storage modulus *E´* (MPa) and damping factor *tan δ* for the composites A, B and C in the initial state

- *MHV*: The highly filled composite A showed the highest values (median 75.1 MHV (*IQR* = 16.3 MHV)). Composite B had a median of 42.8 MHV (*IQR* = 4.8 MHV). The low-filled composite C had a similar median with 43.4 MHV (*IQR* = 6.0 MHV). *MHV* of composite A differed significantly (*p* < 0.05) from composites B and C. In contrast, the median values of composites B and C showed similar values (*p* > 0.05). *MHV* correlated significantly with a rising filler content (*r*_*s*_ = 0.738;* p* < 0.05).
- *σ*_max_: In the initial state, the highly filled composite A reached a median of 408.5 MPa with an *IQR* of 68.3 MPa. Composite B had a median of 327.9 MPa (*IQR* = 46.5 MPa). The low-filled composite C showed a median of 332.7 MPa (*IQR* = 28.6 MPa). In comparison, the median of composite A was 19.7% higher than composite B and 18.6% higher than composite C, but did not differ significantly (*p* > 0.05). *σ*_max_ showed no correlation to filler content (*r*_*s*_ = 0.435;* p* = 0.105).
- *E*_*C*_: The highly filled composite A had the highest values between 5.44 and 6.91 GPa with a median of 6.35 GPa (*IQR* = 0.41 GPa) and differed significantly (*p* < 0.01) from the composites B and C. Composite B had a median of 2.21 GPa (*IQR* = 0.31 GPa). Its values did not differ significantly (*p* > 0.05) from those of composite C, which showed a median of 1.96 GPa (*IQR* = 0.33 GPa) in the initial state. *E*_*C*_ correlated significantly with rising filler content (*r*_*s*_ = 0.850;* p* < 0.01).
- *DMTA*: At the initial state, *E´* and *tan* *δ* were measured at the temperatures at 5 °C, 37 °C, 55 °C and 85 °C and the *Tg* were determined. *E´* of the highly filled composite A reached median values between 4.1 and 4.8 GPa (*n* = 60). The medium-filled composite B had an *E´* with median values between 4.4 and 10.1 GPa. The median values of the low-filled composite C were between 2.8 and 3.5 GPa. *E´* of all composites decreased with rising temperature and with increasing frequency (Fig. 5). Exemplarily, the highly filled composite A showed the lowest decrease from 5 to 85 °C for *E´*. The medium-filled composite B and the low-filled composite C behaved analogously. Comparing the three different composite groups, they differed significantly (*p* < 0.05). The highly filled composite A had a 25.7% higher *E´* than the low-filled composite C at a frequency of 1 Hz and a temperature of 37 °C. *E´* of the composite C was higher than composite A at all measured frequencies and temperatures. The medium-filled composite B had a storage modulus that was approximately twice higher than *E´* of the highly filled composite A. The damping factor *tan δ* as the quotient of *E´´ and E´* was considered in a second analysis. *Tan* *δ* was accordingly contrary to *E´*. Under thermal treatment, the composites showed an increasing *tan δ* leading to a loss in damping. Initially, it was for the highly filled composite A at 0.026 at a frequency of 1 Hz and changed with higher temperature to 0.090. Damping factor of the medium-filled composite B showed values in the range between 0.031 and 0.222. The low-filled composite C had values in the range between 0.018 and 0.177.

*Tg* was calculated by the maximum of *tan δ*. This maximum was determined for each of the composites depending on frequency. At the initial state, median value of *Tg* of the highly filled composite A was 126.0 °C (*IQR* = 2.1 °C) at a frequency of 0.5 Hz, for composite B at 99.6 °C (*IQR* = 5.0 °C) and for composite C at 121.8 °C (*IQR* = 4.0 °C). In comparison, composites A and B and composites B and C differed significantly (*p* < 0.01). In dependence of rising frequencies, a shift of *Tg* to higher temperatures occurred. At a frequency of 1 Hz, *Tg* of the highly filled composite increased by 0.4%, the medium-filled by 3.7% and the low-filled by 4.4%. With a further increase to a frequency of 3.3 Hz, *Tg* also increased.

### Effects of chemical degradation on composite characteristics

To analyze the effects of degradation media (artificial saliva, lactic acid, citric acid and ethanol) and time, specimens were stored in the various media and static, dynamic and physicochemical parameters were measured after 14, 30, 90 and 180 days.

**Fig.6:**
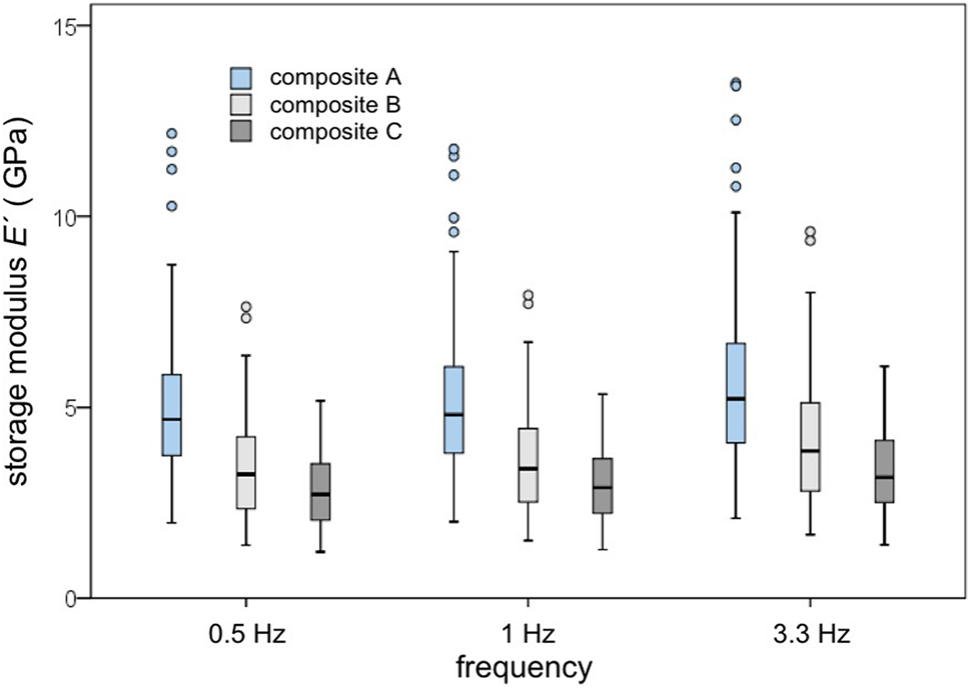
Frequency dependency of storage modulus: *E´* (GPa) of the composites A, B and C after 180 days (in order to determine the influence of time, data of different media were summarized)

- *MHV: MHV* of all composites was reduced over a period of 180 days. For artificial saliva, decrease was not significant (*p* > 0.05). Lactic acid and citric acid provided similar results (Table 2). Storing the specimens of composite C in ethanol, *MHV* decreased without any statistical significance (*p* > 0.05). Storage in ethanol showed different data. Ethanol reduced *MHV* of all composites, but in different ways. Highest loss was observed for the low-filled composite from 43.4 MHV (*IQR* = 6.0 MHV) to 29.1 MHV (*IQR* = 2.3 MHV) after 180 days (*p* < 0.05) which means a reduction by 33%. In contrast, *MHV* remained highest for the highly filled composite A (62.4 MHV; *IQR* = 7.1 MHV) which means a reduction by only 17%.
- *σ*_max_: All composites showed no significant decrease for artificial saliva (*p* > 0.05). Exemplarily, median value of the highly filled composite A decreased from 408.5 MPa (*IQR* = 68.3 MPa) to 334.8 MPa (*IQR* = 131.3 MPa). In comparison, composite B showed a median of 238.6 MPa (*IQR* = 78.9 MPa) in the same aging medium after 180 days. Lactic acid and citric acid showed similar results. Ethanol reduced *σ*_max_ of the different composites the most, but only *σ*_max_ of composite C showed a significant (*p* < 0.05) decrease from 332.7 MPa (*IQR* = 28.6 MPa) to 275.3 MPa (*IQR* = 12.3 MPa) .
- *E*_*C*_: Artificial saliva showed no influence to all composites (*p* > 0.05). Composite A showed the highest measured values, followed by composites B and C. Lactic acid and citric acid had a greater influence on *E*_*C*_ than artificial saliva in all composites without statistical significance (*p* > 0.05). Exemplarily, *E*_*C*_ of composite A decreased by 12.4% due to artificial saliva, by 19.0% due to lactic acid and by 30.5% due to citric acid. However reduction was not significant (*p* > 0.05). Ethanol reduced *E*_*C*_ of the three composites the most, whereby only the low-filled composite C showed a significant decrease (*p* < 0.05). The highly filled composite A repeatedly showed the highest *E*_*C*_ with a median value of 4.49 GPa (IQR = 1.46 GPa) after 180 days due to ethanol storage.
- *DMTA: E´* tended to decrease in the range from 5 to 85 °C (Table 3). The highly filled composite A showed nearly no changes for *E´* for artificial saliva (*f* = 1 Hz, *t* = 37 °C). In comparison, the medium-filled composite B had a decrease by approximately 50%. The values of *E´* of composite B stored in artificial saliva increased by 55.8% after 180 days. Composite B stored in lactic acid and citric acid showed similar results. *E'* of composite B stored in ethanol showed the highest decrease by 74.5% after 180 days (*f* = 1 Hz, *t* = 37° C). With regard to the filler content, all composites behaved similarly to the initial state. Figure 6 shows exemplarily the frequency dependency of *E´* of the composites A, B and C after 180 days.

**Table 2:** Results of static parameters in comparison: *MHV* (MHV), *σ*_max_ (MPa), *E*_*C*_ (GPa). Values given as median (IQR). *p < 0.05

	Storage medium	MHV (MHV) initial state	MHV (MHV) after 180 days	*σ*_max_ (MPa) initial state	*σ*_max_ (MPa) after 180 days	*E*_*C*_ (GPa) initial state	*E*_*C*_ (GPa) after 180 days
Composite A	Control	75.1 (16.3)		408.5 (68.3)		6.36 (0.41)	
	Artificial saliva		92.4 (4.9)		334.8 (131.3)		5.57 (1.84)
	Lactid acid		100.2 (8.8)		308.7 (162.4)		5.15 (2.90)
	Citric acid		93.3 (30.8)		265.7 (45.1)		4.42 (1.34)
	Ethanol		62.4 (7.1)		301.8 (104.3)		4.49 (1.46)
Composite B	Control	42.8 (4.8)		327.9 (46.5)		2.21 (0.31)	
	Artificial saliva		45.3 (6.9)		238.6 (78.9)		2.11 (0.24)
	Lactid acid		44.1 (7.0)		248.9 (50.4)		1.85 (0.78)
	Citric acid		39.6 (7.0)		291.9 (14.1)		1.86 (0.10)
	Ethanol		39.4 (6.1)		142.9 (39.7)		1.78 (0.41)
Composite C	Control	43.4 (6.0)		332.7 (28.6)		1.96 (0.33)	
	Artificial saliva		33.9 (2.9)		345.7 (60.5)		1.95 (0.02)
	Lactid acid		29.3 (3.6)		356.4 (66.1)		1.61 (0.28)
	Citric acid		32.5 (2.8)		283.6 (59.9)		1.64 (0.20)
	Ethanol*		29.1 (2.3)*		275.3 (12.3)*		1.59 (0.10)*

**Table 3:** Results of dynamic compression tests in comparison: *E´* (GPa). Values given as median (IQR)

		Composite A		Composite B		Composite C	
Storage medium	Temperature (°C)	Initial state	After 180 days	Initial state	After 180 days	Initial state	After 180 days
Control	5	4.5 (0.3)		8.1 (2.8)		3.3 (0.8)	
	37	4.2 (0.4)		8.6 (2.8)		3.1 (0.2)	
	55	4.1 (0.5)		7.9 (1.2)		3.2 (0.6)	
	85	4.4 (0.7)		4.9 (0.6)		3.0 (0.4)	
Artificial saliva	5		4.6 (4.0)		4.3 (0.3)		4.3 (0.3)
	37		4.3 (3.8)		3.8 (1.5)		3.8 (1.5)
	55		4.2 (4.2)		3.4 (0.2)		3.4 (0.2)
	85		4.0 (3.9)		1.6 (0.2)		1.6 (0.2)
Lactid acid	5		7.2 (1.9)		4.3 (1.3)		4.4 (0.7)
	37		5.5 (1.4)		3.9 (0.4)		4.0 (0.6)
	55		5.3 (1.7)		3.2 (0.7)		3.3 (0.8)
	85		4.3 (1.8)		1.6 (0.2)		2.3 (0.4)
Citric acid	5		8.1 (6.4)		5.2 (2.8)		3.3 (0.8)
	37		5.6 (4.8)		3.8 (1.2)		2.9 (0.4)
	55		6.7 (6.2)		3.4 (0.7)		2.3 (0.5)
	85		6.2 (3.2)		1.5 (0.2)		1.5 (0.2)
Ethanol	5		6.9 (0.5)		4.6 (2.3)		4.1 (0.9)
	37		4.8 (1.1)		2.2 (0.7)		2.2 (0.3)
	55		4.7 (0.9)		1.7(0.3)		1.6 (0.2)
	85		4.2 (0.4)		1.2 (0.1)		1.1 (0.1)

*Tan δ* was also considered in relation to the initial state. Damping factor showed discontinuous values similar to storage modulus curves. Overall, the composites showed a *tan δ* < 0.2 over time under the influence of the media.

*Tg* can be evaluated as a direct indicator for polymer aging processes. It dropped sharply for specimens stored in ethanol over time and decreased without any statistical significance (*p* > 0.05) by 17.1% after 180 days. *Tg* of samples stored in citric acid also decreased without any statistical significance (*p* > 0.05) by 8.3%. Similar results were obtained for the composites stored in artificial saliva (6.1%) and lactic acid (5.4%). When these data were considered differently according to the filler content, the highly filled composite A had the highest glass transition temperature after 180 days.

In order to determine the influence of time, all media were summarized for one point in time. Glass transition temperature dropped after 14, 30, 90 and 180 days. Even at higher frequencies, the glass transition temperature decreased compared to its initial value.

- *Water sorption and solubility:* For composite A, no statistically significant difference was found between water sorption after 14 and 180 days for all media (*p* > 0.05) (Table 4). In contrast, the test specimens of the composites B and C stored in lactic acid and ethanol showed a statistically significant (*p* < 0.05) change in water sorption comparing the results at the start (14 days) with the results at the end of the tests (180 days). With increasing filler content, water sorption decreased. (*r*_*s*_ =  − 0.506;* p* < 0.01). Solubility did not correlate significantly with filler content (*r*_*s*_ = 0.177;* p* = 0.388). For the composite A, no statistically significant decrease was found for all media. The test specimens of composites B and C stored in lactic acid and ethanol differ significantly (*p* > 0.05) (Table 4). Ethanol caused the highest solubility after 180 days.

**Table 4:** Results of physicochemical parameters in comparison: water sorption and solubility. Values given as median (IQR) *p < 0.05

	Storage medium	Water sorption (µg mm^−3^) after 14 days	Water sorption (µg mm^−3^) after 180 days	Solubility (µg mm^−3^) after 14 days	Solubility (µg mm^−3^) after 180 days
Composite A	Artificial saliva	16.5 (16.5)	44.8 (22.0)	20.4 (12.3)	35.8 (21.9)
	Lactid acid	18.7 (17.1)	37.6 (11.1)	9.3 (13.0)	41.1 (19.0)
	Citric acid	28.6 (28.5)	27.5 (15.9)	19.1 (20.4)	19.4 (7.4)
	Ethanol	18.6 (5.9)	41.6 (44.4)	29.9 (13.4)	62.9 (87.0)
Composite B	Artificial saliva	27.4 (21.6)	48.7 (58.9)	17.8 (4.3)	25.3 (12.0)
	Lactid acid*	13.0 (7.9)	68.6 (31.6)*	8.1 (8.0)	39.2 (4.1)*
	Citric acid	31.9 (10.4)	72.9 (22.9)	16.3 (8.2)	40.5 (32.0)
	Ethanol*	18.0 (7.0)	118.0 (12.0)*	24.0 (13.5)	55.1 (20.5)*
Composite C	Artificial saliva	35.7 (20.4)	71.8 (12.0)	8.8 (8.9)	26.9 (19.8)
	Lactid acid*	28.9 (14.0)	61.2 (12.2)*	9.4 (4.8)	38.5 (18.3)*
	Citric acid	29.7 (14.4)	45.9 (19.5)	9.9 (9.4)	38.0 (14.0)
	Ethanol*	28.5 (8.4)	82.9 (21.9)*	9.0 (9.0)	44.9 (51.7)*

## Discussion

Aim of the present in vitro study was to determine factors influencing the degradation of *DC* by simulating a natural oral environment. For this purpose, three commercially available *DC* (GrandioSo ®, Arabesk Top®, Arabesk Flow®) with different filler contents were subjected to chemical degradation media to examine their mechanical and physicochemical properties before and after treatment. Every composite was selected with respect to their filler content as a representative example for classification into highly (> 80% by weight), medium- (71–79% by weight) and low-filled (< 70% by weight) composites. The filler content is one of the many possible ways in which composites can be classified. Another type of classification is the subdivision according to consistency into packable materials as well as flowables. Nevertheless, the mechanical properties of packable composites are extremely different depending on the filler content. There are many in vivo studies that proved the use and longevity of composites in the posterior region through clinical evaluation without mechanical tests after wearing. The conclusion of a meta-analysis of Opdam et al. [31] including 12 clinical studies showed that posterior resin composite restorations show a good survival with annual failure rates of 1.8% at 5 years and 2.4% after 10 years of service. Rocha Gomes Torres et al. [32] evaluated clinical performance of class II restorations made with composite with high filler content and different consistency in a 2-year study. No significant differences were observed between GrandioSO® conventional and GrandioSO Heavy Flow®. In 2015, an in vitro long-term study by Alshali et al. [33] including GrandioSO® also showed that water sorption and solubility of resin-composites are highly affected by the filler loading. Disc shaped samples (*n* = 5) were randomly immersed into distilled water and artificial saliva for 1-year period and weighed at different time intervals. MacAulay et al. [34] tested in vivo degradation of bisGMA and urethane-modified bisGMA-based resin composite materials. They proved that composites did not release any detectable amount of bisGMA breakdown products. In contrary, Dedy Kusuma Yulianto et al. [35] showed in an in vivo study that a high ester-linkage composite (Beautifill II®, highly filled composite with 83w%) harvested oral biofilm that more frequently possesses *S. mutans* and was subject to stronger intraoral degradation regardless of oral hygiene regime applied when compared to a low ester-linkage composite. Therefore, volunteers were equipped with a palatal appliance containing cavities with discs of two different composites. For the present study, a very small cuboid specimen geometry was used for static and dynamic compression tests to better transfer the results to the in vivo situation where the dental hard tissue differs in their mechanical behavior in a small scale with regard to the tooth area and ultrastructure.

### Methodological considerations

Specimen geometry is the reason why it is crucial to compare studies, because it varies enormously in existing publications. Discs [12] and sticks [13] with different diameters and dimensions were used. In addition, the test modes vary, too. In accordance to DIN EN ISO [23], a bar-shaped geometry is recommend which allows only the measurement of bending properties. In posterior regions, compression loads dominate, but they are neglected until now. Therefore, for the present study, cuboid samples were chosen, which were commonly used in testing of enamel and dentine [14, 36]. Thus, a standardized test procedure is missing. Since dentine and enamel occurred only in small layer thickness, a test specimen geometry with very small dimensions is recommended for direct comparison. Previous tests showed that dentine and enamel do not overlap with this size. For this purpose, healthy, extracted teeth were sawn into cubes of different sizes and examined microscopically to determine whether enamel, dentine or a mixture of both was present. Specimen geometry of present study was chosen, to enable a comparison of results with dentin and enamel, which also have different mechanical properties.

Zaytsev et al. [37] postulated that the mechanical properties of dentine depend on the geometry of specimen. The experiments for dentine have shown that it is a practically undeformed brittle substance under bending when considerable tensile stress is applied to the sample. In contrast under compression (when the level of tensile stress in a sample is considerably lower), dentine shows various types of deformation behavior, which depends on the diagonal compression surface and the height of cuboid sample (d/h ratio) [36]. The result is a lower *E*_*C*_ under compression than under bending. In a direct comparison, Warkentin and Sabbagh et al. [14, 38] also confirmed these results for composites. Dentin samples can exhibit considerable elasticity as well as plasticity under high compression (up to 800 MPa). The experiments of Zaytsev et al. [36] showed a strong dependence on the d/h ratio. He named his type of deformation behavior “shape effect”. Dentin samples with a low aspect ratio behave as an almost brittle solid, whereas the samples with a high aspect ratio show high elasticity and plasticity [36].

Due to the sample size, the different time points and various media, a number of 807 test specimens were examined. Specimens of three commercially available composites were stored in four media and measured in their initial state and after 14, 30, 90 and 180 days. Vickers microhardness (*n* = 3 for each parameter, composite group, time point and media) was determined of 153 specimens. For 255 specimens, the determination of the compressive strength and the elastic modulus were carried out by quasi-static pressure tests (*n* = 5 for each parameter, composite group, time point and media). A total of 255 specimens were characterized with compression tests with dynamic-mechanical-thermal analysis. To determine the physicochemical parameters, the water sorption and the solubility of 144 specimens were determined (*n* = 3 for each parameter, composite group, time point and media). In any case, it has to be mentioned that the choice of only three composites limits conclusions of the study and further studies would be desirable. The development of structure compatible and functionally adapted composites requires further research and maybe new filling concepts. Accordingly, the use of compression tests to determine values for developing structure compatible and functionally adapted composites, especially for posterior regions where compression load dominates, makes sense as an additional standard and was used in this study. In addition to the used cuboid samples, a different surface-to-volume ratio resulted therefrom. Accordingly, in literature, deviations in terms of the values were found and the results were difficult to compare. Moreover, DIN EN ISO 4049 [23] recommends a water immersion and gravimetric observation after 7 days with a resulting solubility of less than 7.5 µg mm^−3^. In this experimental setup, the first sampling date started after 14 days. Hence, solubility was higher than the limit even after 14 days. It is quite possible that artificial saliva, and lactic and citric acid will reach standard values after 7 days. Therefore, further investigations are needed. On the other hand, solubility is influenced by surface area and specimen volume. Measurements in accordance with the DIN standard [23] used test specimens in form of discs with a diameter of 15 mm and a height of 1 mm. In comparison, the surface-area-to-volume-ratio of the cubes used in this study was 2.6 times higher than the ratio of the discs of the DIN standard. Therefore, the quantitatively higher results are probably caused by the deviating ratio. Additionally, the ISO 4049 standard defines the conditions for performing tests of composites to allow reproducibility [39]. Nevertheless, the alternative use of a light-curing device in this study enables very uniform curing and, in contrast to a polymerization lamp, prevents overlaps in the exposed areas, so that better reproducibility is given.

### Influence of filler content on composite characteristics

Filler content had a great impact on the static mechanical parameters *MHV* and *E*_*C*_. In contrast to *σ*_max_, these parameters correlated (*p* < 0.05) with the filler content even after 180 days of storage. As other studies have shown [40, 41], *MHV* increased with rising filler content in the initial state. Composite A showed significantly higher *MHV* than the other composites. These may be due to the incorporated silanized fillers. In conclusion of specimens stored in ethanol, the highly filled composite A also had the highest median values after 180 days of storage and should therefore preferentially be used for occlusal fillings. *MHV* of enamel (270 to 420 HV) or dentine (10 to 90 HV) [14, 42] is significantly higher than *MHV* of the tested composites. Accordingly, an enhanced wear of the composites is expected in vivo by additional chemical degradation (see below). Thus, a uniform abrasion behavior of tooth substance and filling material would be desirable which may only be reached by new filler-matrix-concepts or graded materials [43].

*σ*_max_ has also a great relevance of *DC* performance in vivo, because compression is particularly important in the posterior region, in which composites are increasingly being used today. In this study, it could be shown that *σ*_max_ increased with an increasing filler content. This correlation is confirmed by the literature [44].

Another important aspect of this study was the determination of *E*_*C*_. According to the literature, most medium- to highly filled composites have an *E*_*C*_ with maximum values of 15 GPa and thus, low-filled composites showed lower results [44]. These results could not be confirmed in the present study. For the highly filled composite A, the highest median value was found with 6.36 GPa (IQR = 0.41 GPa). These deviations result primarily from selected specimen geometry and the used test modes. Regardless of the experimental setup and procedure, all studies showed that *E*_*C*_ increases with filler content, which can be confirmed by the present work [14, 44]. As for all the static parameters mentioned, the test specimen geometry is decisive for the results. Therefore, a comparison with the current literature is problematic.

Similar results were shown for dynamic parameters. After 180 days, the highly filled composite A had the highest *E'* independent from media and frequencies used. The lowest *E'* had the low-filled composite C. Exemplarily, the composite A showed median values between 4.3 and 5.6 GPa after 180 days in the different media (*f* = 1 Hz, *t* = 37° C), while composite C reached median values between 2.2 and 4.0 GPa. There is currently no published literature on compression tests using *DMTA* on dental composites. Anyway, bending tests were used and degradation time was 30 and 90 days, respectively. In these studies [45, 46], test specimens were stored in dry air, distilled water, artificial saliva, heptanes and ethanol (75%). Medium-filled and highly filled composites were assessed at a frequency of 1 Hz. In case of anisotropic composites, values depend on the measurement mode. Accordingly, shear, tensile and compression measurements are preferable to the bending measurements [5]. In relation to the filler content, comparable composites achieved higher values in the three-point bending test than in our own test series. The higher energy consumption and storage under dynamic load using this measuring method can also be explained by the different points of application of the same force compared to the compression test, which results in a higher *E'* and a higher damping factor in the three-point bending test. In accordance with the literature [14, 47, 48], the own results show that, if temperature increased, *E'* decreased and *tan δ* increased. It was demonstrated that percentaged decrease in *E'* is influenced by the filler content. Overall, the highly filled composite A had a much lower decrease in *E'* than the other two composites in the same temperature ranges. A filler-dependent reduction in *E'* was also detected for the three frequencies examined. The medium-filled composite B shows a special feature. It looked like a highly filled composite in the initial state and had a higher *E'* than composite A. Morphological studies in our own working group indicated that the incorporated fillers form agglomerates and show properties of a highly filled material. Molecular side chains are restricted in their mobility at low temperatures. As temperature rises, molecular chain mobility increases [29] and polymer volume increases. If temperature continues to rise, the polymer begins to soften up to thermal decomposition and the result is a sinking *E'*. The incorporation of fillers leads to a shift in this sequence to higher temperatures. The own results show that incorporation of fillers with more than 80 w% leads to significantly better mechanical properties under dynamic compression.

*Tg* was examined on one hand to characterize the influence of the filler-matrix-concept in relation to rising filler content and on the other hand to characterize the polymer aging process by the shift of *Tg*. Therefore, *Tg* corresponds to the maximum loss factor. It decreased after 180 days for all test specimens (*p* > 0.05). In addition, *Tan δ* was below 0.2 at the end of the test series for all composites, which indicates a predominantly elastic behavior and, thus, a very good functionality. However, one must take into account that *DMTA* represents a mechanical *Tg* influenced by filler and matrix. In contrast, a chemical *Tg* (tested with differential scanning calorimetry-DSC) would only reflect properties of the polymer itself. Thus, the matrix corresponding *Tg* will be lower (around 50 °C) and shifts due to aging may be more pronounced.

With regard to the physicochemical parameters, water sorption changes for composite A were not statistically significant (for all media) comparing the beginning and day 180 (*p* > 0.05). In contrast, the test specimens of composites B and C stored in lactic acid and ethanol showed a statistically significant (*p* < 0.05) change in water sorption comparing the results at the start (14 days) with the results at the end of the tests (180 days). It was found a significant correlation with filler content (*r*_*s*_ =  − 0.506;* p* < 0.01). After 14 days, all assessed composites had a water sorption of less than 40 µg mm^−3^ according to DIN EN ISO 4049 [23] after 7 days. Solubility was not significantly correlated with filler content (*r*_*s*_ = 0.177;* p* = 0.388).

Despite the existence of a similar matrix, the assessed composites did not show identical degradation behavior. Composite A has a higher filler content than the other two composites. As a result, it shows better mechanical properties in the initial state and after degradation. This difference in the extent of degradation can be explained by the fact that a higher filler content leads to a reduced diffusion and limits the total extent of effective degradation media [49]. Schwartz and Söderholm postulated in 2004 [50] that the use of more voluminous filler particles in dental composites also resulted in an increased reduction in mechanical properties over time due to the increased space between molecules. In case of the highly filled composite A, this effect can be prevented by embedding large micro-fillers and smaller nano-fillers, which is confirmed by the present study. In addition to filler content, the filler type is also important.

### Influence of degradation media and time on composite characteristics

In contrast to filler content, results show that the influence of degradation media and time play an underpart role for degradation. Only a few statistically significant results were available for static parameters. Due to storage in various media, only *MHV*, *σ*_max_ and *EC*of the low-filled composite C were reduced significantly by ethanol (*p* < 0.05). Numerous studies have shown that artificial aging leads to a reduction in mechanical properties of *DC* [12, 51, 52]. The connecting element (silanization) between the inorganic and organic phases is weakened by hydrolytic chain splitting [9]. The effect of acids on microhardness is controversially discussed in literature. There are studies that show a decrease in *MHV* due to lactic acid [53], citric acid [54] or acidic foods [55]. Other studies that show an increase in microhardness [25] or no change with lactic acid [10] or citric acid [53]. This study also showed discontinuous values in test periods for artificial saliva, lactic acid and citric acid. In addition, this study confirmed that ethanol leads to a reduction in *MHV* [27]. Considering the influence of media, similar results can be found in the literature for *σ*_max_ [56] and *E*_*C*_ [56]. As shown for static parameters, ethanol also caused the most significant reduction in the dynamic parameters, which results in a decreasing *E'* (*p* > 0.05) and is confirmed by Vouvoudi et al. [19, 45] and Sideridou et al. [46]. The highly filled composite A provided the highest *E'* for all media after 14, 30, 90 and 180 days. *Tg* tended to decrease for all media (*p* > 0.05). In comparison to initial state, *Tg* of specimens stored in ethanol dropped the most after 180 days *(p* > 0.05*)*. Physicochemical parameters showed that test specimens of composites B and C stored in lactic acid and ethanol decrease significantly (*p* < 0.05). Changes in water sorption present neither a continuous reduction nor a continuous increase over time. Ethanol caused the highest solubility after 180 days. The literature describes that ethanol has a more aggressive potential and causes higher water sorption and solubility than water or artificial saliva [57]. It should be emphasized again that comparison with other studies is difficult as specimen geometry, the selected media, the composition of media and the examination periods used in this study differ from the experimental setups in the literature.

## Conclusion

Although composites have similar matrix, they did not show identical degradation behavior. Incorporation of fillers with more than 80 w% leads to significantly better mechanical properties under static and dynamic compression tests and a better water sorption behavior even after chemical degradation. A preferential use of highly filled composites for occlusal fillings is recommended and of clinical relevance for the longevity of the composite restorations. Here, very elastic materials with a low *E*_*C*_ would give way due to deformation and might even damage the surrounding dental hard tissue. Indeed, the use of low-filled composites as fissure sealing to prevent cavities should be reconsidered. A combination of composites with different filler content for direct restorations appears to be useful. In addition, the use of compression tests in order to determine material characteristics (especially in small scale) would be helpful for developing compatible and functionally adapted composites and should be considered to be established as an additional standard.

## References

[1] Torres CRG, Schwendicke F (2020) General principles of tooth preparation and carious tissue removal. In: modern operative dentistry. Springer, pp 183–221

[2] Mjör IA, Dahl JE, Moorhead JE (2000). Age of restorations at replacement in permanent teeth in general dental practice. Acta Odontol Scand.

[3] Sadava DE, Hillis DM, Heller HC, Berenbaum M (2011). Biologie. 9. Auflage edn.

[4] Ehrenstein GW (2010). Polymer-Werkstoffe: Struktur - Eigenschaften - Anwendung. 3., vollständig überarbeitete Auflage edn.

[5] Grellmann W, Altstädt V (2011). Kunststoffprüfung. 2. Aufl. edn.

[6] Cai K, Delaviz Y, Banh M, Guo Y, Santerre JP (2014). Biodegradation of composite resin with ester linkages: identifying human salivary enzyme activity with a potential role in the esterolytic process. Dent Mater.

[7] Finer Y, Santerre JP (2003). Biodegradation of a dental composite by esterases: Dependence on enzyme concentration and specificity. J Biomater Sci Polym Ed.

[8] Göpferich A (1996). Mechanisms of polymer degradation and erosion. Biomater.

[9] Koin PJ, Kilislioglu A, Zhou M, Drummond JL, Hanley L (2008). Analysis of the degradation of a model dental composite. J Dent Res.

[10] Münchow EA, Ferreira ACA, Machado RMM, Ramos TS, Rodrigues-Junior SA, Zanchi CH (2014). Effect of acidic solutions on the surface degradation of a micro-hybrid composite resin. Braz Dent J.

[11] Sideridou ID, Achilias DS, Karabela MM (2007). Sorption kinetics of ethanol/water solution by dimethacrylate-based dental resins and resin composites. J Biomed Mater Res Part B, Applied biomaterials.

[12] Krüger J, Maletz R, Ottl P, Warkentin M (2018). In vitro aging behavior of dental composites considering the influence of filler content, storage media and incubation time. PloS one.

[13] Belli R, Geinzer E, Muschweck A, Petschelt A, Lohbauer U (2014). Mechanical fatigue degradation of ceramics versus resin composites for dental restorations. Dent Mater.

[14] Warkentin M (2014). Werkstoffkundliche und strukturmorphologische Charakterisierung von Zahnhartsubstanzen und dentalen Füllungskompositen. Habilitation Thesis.

[15] Krejci I, Reich T, Lutz F, Albertoni M (1990). In-vitro-Testverfahren zur Evaluation Dentaler Restaurationssysteme. 1. Computergesteuerter Kausimulator (An in vitro test procedure for evaluating dental restoration systems. 1. A computer-controlled mastication simulator). Schweiz Monatsschr Zahnmed.

[16] DeLong R, Douglas WH (1983). Development of an artificial oral environment for the testing of dental restoratives: bi-axial force and movement control. J Dent Res.

[17] Corica A, Caprioglio A (2014). Meta-analysis of the prevalence of tooth wear in primary dentition. Eur J Paediatr Dent.

[18] Li Y, Carrera C, Chen R, Li J, Lenton P, Rudney JD, Jones RS, Aparicio C, Fok A (2014). Degradation in the dentin-composite interface subjected to multi-species biofilm challenges. Acta Biomater.

[19] Vouvoudi EC, Sideridou ID (2013). Effect of food/oral-simulating liquids on dynamic mechanical thermal properties of dental nanohybrid light-cured resin composites. Dent Mater.

[20] Drummond JL (2008). Degradation, fatigue, and failure of resin dental composite materials. J Dent Res.

[21] Ferracane JL, Berge HX, Condon JR (1998). In vitro aging of dental composites in water?: Effect of degree of conversion, filler volume, and filler/matrix coupling. J Biomed Mater Res.

[22] Bouillaguet S (2004). Biological risk of resin-based materials to the dentin-pulp complex. Critical reviews in oral biology and medicine. Crit Rev Oral Biol Med.

[23] DIN EN ISO 4049: Dentistry - polymer-based restorative materials (ISO 4049:2009); German version EN ISO 4049:2009. 4049. Beuth Verlag GmbH, Berlin

[24] McKinney JE, Wu W (1985). Chemical softening and wear of dental composites. J Dent Res.

[25] Yap AU, Tan SH, Wee SS, Lee CW, Lim EL, Zeng KY (2001). Chemical degradation of composite restoratives. J Oral Rehabil.

[26] Wu W, McKinney JE (1982). Influence of chemicals on wear of dental composites. J Dent Res.

[27] Sevimay M, Yucel MT, Tak O (2008) Influence of food simulation solutions on the hardness of composite resins. J Compos Mater. 10.1177/0021998307086205

[28] DIN EN ISO 604: Plastics - determination of compressive properties (ISO 604:2002); German version EN ISO 604:2003. 604. Beuth Verlag, Berlin

[29] Schmiedel H (1992) Handbuch der Kunststoffprüfung. C. Hanser, München

[30] Ehrenstein GW, Riedel G, Trawiel P (2003). Praxis der thermischen Analyse von Kunststoffen. 2., völlig überarb. Aufl. edn.

[31] Opdam NJ, van de Sande FH, Bronkhorst E, Cenci MS, Bottenberg P, Pallesen U, Gaengler P, Lindberg A, Huysmans MC, van Dijken JW (2014). Longevity of posterior composite restorations: a systematic review and meta-analysis. J Dent Res.

[32] Rocha Gomes Torres C, Rego HM, Perote LC, Santos LF, Kamozaki MB, Gutierrez NC, Di Nicolo R, Borges AB (2014). A split-mouth randomized clinical trial of conventional and heavy flowable composites in class II restorations. J Dent.

[33] Alshali RZ, Salim NA, Satterthwaite JD, Silikas N (2015). Long-term sorption and solubility of bulk-fill and conventional resin-composites in water and artificial saliva. J Dent.

[34] MacAulay M, Tam LE, Santerre JP, Finer Y (2017). In Vivo Biodegradation of bisGMA and Urethane-Modified bisGMA-Based Resin Composite Materials. JDR Clin Trans Res.

[35] Kusuma Yulianto HD, Rinastiti M, Cune MS, de Haan-Visser W, Atema-Smit J, Busscher HJ, van der Mei HC (2019). Biofilm composition and composite degradation during intra-oral wear. Dent Mater.

[36] Zaytsev D, Ivashov AS, Mandra JV, Panfilov P (2014). On the deformation behavior of human dentin under compression and bending. Mater Sci Eng C Mater Biol Appl.

[37] Zaytsev D, Grigoriev S, Panfilov P (2012). Deformation behavior of human dentin under uniaxial compression. Inter J Biomater.

[38] Sabbagh J, Vreven J, Leloup G (2002). Dynamic and static moduli of elasticity of resin-based materials. Dent Mater.

[39] Özcan C, Lestriez P, Berry-Kromer V, Thiebaud F, Sockalingum GD, Untereiner V, Angiboust JF, Josset Y (2020). Misinterpretation of ISO 4049 standard recommendations: impact on Young’s modulus and conversion degree of dental composites. J Mech Behav Biomed Mater.

[40] Braem M, Finger W, van Doren VE, Lambrechts P, Vanherle G (1989). Mechanical properties and filler fraction of dental composites. Dent Mater.

[41] Kim K-H, Ong JL, Okuno O (2002). The effect of filler loading and morphology on the mechanical properties of contemporary composites. J Prosthet Dent.

[42] Craig R, PEYTON FA,  (1958). The micro-hardness of enamel and dentin. J Dent Res.

[43] Zisis T, Kordolemis A, Giannakopoulos AE (2010). Development of strong surfaces using functionally graded composites inspired by natural teeth—finite element and experimental verification. J Eng Mater Technol.

[44] Masouras K, Silikas N, Watts DC (2008). Correlation of filler content and elastic properties of resin-composites. Dent Mater.

[45] Vouvoudi EC, Sideridou ID (2012). Dynamic mechanical properties of dental nanofilled light-cured resin composites: effect of food-simulating liquids. J Mech Behav Biomed Mater.

[46] Sideridou ID, Vouvoudi EC, Adamidou EA (2015). Dynamic mechanical thermal properties of the dental light-cured nanohybrid composite Kalore, GC: effect of various food/oral simulating liquids. Dent Mater.

[47] Mesquita RV, Axmann D, Geis-Gerstorfer J (2006). Dynamic visco-elastic properties of dental composite resins. Dent Mater.

[48] Sideridou ID, Karabela MM, Vouvoudi EC (2008). Dynamic thermomechanical properties and sorption characteristics of two commercial light cured dental resin composites. Dent Mater.

[49] Oysaed H, Ruyter IE (1986). Water sorption and filler characteristics of composites for use in posterior teeth. J Dent Res.

[50] Schwartz JI, Söderholm K-JM (2004). Effects of filler size, water, and alcohol on hardness and laboratory wear of dental composites. Acta Odontol Scand.

[51] Catelan A, Briso ALF, Sundfeld RH, Dos Santos PH (2010). Effect of artificial aging on the roughness and microhardness of sealed composites. J Esthet Restor Dent.

[52] Hahnel S, Henrich A, Bürgers R, Handel G, Rosentritt M (2010). Investigation of mechanical properties of modern dental composites after artificial aging for one year. Oper Dent.

[53] Chadwick RG, Mccabe JF, Walls AWG, Storer R (1990). The effect of storage media upon the surface microhardness and abrasion resistance of three composites. Dent Mater.

[54] Mohamed-Tahir MA, Tan HY, Woo AAS, Yap AUJ (2005). Effects of pH on the microhardness of resin-based restorative materials. Oper Dent.

[55] Erdemir U, Yildiz E, Eren MM, Ozel S (2012). Surface hardness of different restorative materials after long-term immersion in sports and energy drinks. Dent Mater J.

[56] Nouri M-R (2003). Comparison of the fracture toughness, flexural strength, and flexural modulus of nine restorative materials over five time intervals.

[57] Sideridou ID, Vouvoudi EC, Keridou IV (2015). Sorption characteristics of oral/food simulating liquids by the dental light-cured nanohybrid composite Kalore GC. Dent Mater.

